# PINK1-Mediated Mitochondrial Activity Confers Olaparib Resistance in Prostate Cancer Cells

**DOI:** 10.1158/2767-9764.CRC-24-0339

**Published:** 2024-11-20

**Authors:** Zachary A. Schaaf, Shu Ning, Amy R. Leslie, Masuda Sharifi, Richard Y. Gao, James P. Maine, Wei Lou, Alan P. Lombard, Chengfei Liu, Ai-Ming Yu, Nicholas Mitsiades, Allen C. Gao

**Affiliations:** 1 Department of Urologic Surgery, University of California Davis, Davis, California.; 2 Division of Hematology and Oncology, University of California Davis, Davis, California.; 3 Department of Biochemistry and Molecular Medicine, University of California Davis, Davis, California.; 4 UC Davis Comprehensive Cancer Center, University of California Davis, Davis, California.; 5 VA Northern California Health Care System, Sacramento, California.

## Abstract

Olaparib, a PARP inhibitor, is a targeted therapy used in treating various cancers, including castration-resistant prostate cancer. Despite its efficacy, resistance to olaparib remains a significant challenge. Understanding the molecular mechanisms underpinning this resistance is crucial for developing more effective treatment strategies. This study focuses on elucidating the role of mitochondrial alterations and the PTEN-induced kinase 1 (*PINK1*) gene in conferring olaparib resistance in castration-resistant prostate cancer cells. We investigated the transcriptomic and functional differences in mitochondrial activity between olaparib-resistant (2B-OlapR, LN-OlapR) and treatment-naïve prostate cancer cells (C4-2B, LNCaP) in both castration-sensitive and -resistant settings. Through RNA sequencing and gene set enrichment analysis, we identified significant enrichment of mitochondrial activity– and oxidative phosphorylation–related gene sets in olaparib-resistant derived cell lines. Resistant lines exhibited enhanced mitochondrial functionality, including increased basal and maximal respiration rates, as well as elevated ATP production and spare respiratory capacity, compared with parental cells. Subsequent investigations revealed a substantial increase in mitochondrial mass and electron transport chain complex I activity in olaparib-resistant cells. Furthermore, overexpression of the *PINK1* gene was observed in resistant cells, which was correlated with resistance to olaparib and poor clinical outcomes in patients with prostate cancer. Inhibition of PINK1 expression significantly reduced mitochondrial function and mass, impaired cell growth, and decreased resistance to olaparib. These findings suggest that PINK1 plays a crucial role in modulating mitochondrial dynamics that confer therapeutic resistance, highlighting its potential as a therapeutic target for overcoming olaparib resistance in prostate cancer.

**Significance:**

Olaparib, a PARP inhibitor, is effective against various cancers, including prostate cancer. However, resistance to olaparib poses a significant challenge. This study uncovers that mitochondrial alterations and *PINK1* gene overexpression contribute to this resistance in prostate cancer cells. Enhanced mitochondrial functionality and increased PINK1 expression in olaparib-resistant cells underscore the importance of targeting mitochondrial dynamics and PINK1 to develop more effective treatments for overcoming olaparib resistance in prostate cancer.

## Introduction

As the most common organ-specific cancer diagnosis for men, prostate cancer is highly prevalent (1). Initial therapies utilized for treatment which work to reduce systemic androgens such as castration- or androgen production–targeting therapeutics prove to be highly effective initially; however, in as little as just 5 years, almost 20% of patients experience castration-resistant prostate cancer (CRPC; refs. 2–4). Discovery and subsequent use of applicable therapeutics such as next-generation antiandrogen therapies as well as nonandrogen related therapies like taxanes and PARP inhibitors enable effective treatment of patients with CRPC (5). Olaparib, a small-molecule inhibitor with a molecular weight of 435.08 g/mol serves as a potent inhibitor of PARP1 and PARP2, with IC_50_ values of 5 and 1 nmol/L, respectively (6). Inhibition of PARP is observed to cause an accumulation of unrepaired DNA damage in rapidly dividing cancer cells, leading to double-strand breaks which promote cell signaling toward apoptosis (7, 8). As of 2020, olaparib was approved for use in DNA repair–deficient patients with advanced CRPC. In patients harboring mutations in genes essential for homologous recombination, PARP inhibition has proved to be effective across various cancers (9). Recent phase III clinical trials PROfound and PROpel show that olaparib increases progression-free survival in patients with homologous repair–deficient metastatic CRPC (10, 11). As a result of these observations about olaparib response, clinical use is expected to increase.

Unfortunately, various mechanisms of acquired therapeutic resistance in cancer arise, varying between cancer types (5, 12–15). Clinically observed resistance to olaparib has already been noted in patients with cases including mutations that restore functions to DNA repair genes as well as undefined cases in more advanced cancer stages (16–18). In the laboratory setting, olaparib resistance has been generated in human prostate cancer model cell lines (LNCaP and C4-2B) subjected to chronic and increasing dosing of olaparib over a year. The resultant olaparib-resistant lines (Ln-OlapR and 2B-OlapR) are resistant to olaparib treatment compared with parental lines LNCaP and C4-2B, respectively (19, 20). Furthermore, IGFBP3-mediated EGFR activation has been identified to promote resistance to olaparib in both LNCaP and C4-2B cells (21).

In the present study, we determined mitochondrial activity associated with olaparib resistance in the resistant cell lines. RNA extraction and RNA sequencing (RNA-seq) were conducted, and gene set enrichment analysis (GSEA) was performed on the RNA-seq data set. Utilizing the Molecular Signatures Database (MSigDB) Hallmark gene set collection (22), oxidative phosphorylation (OxPhos) was identified as the gene set with the highest enrichment. Subsequent analyses further identified increased expression of sets of genes relating to the production and maintenance of mitochondria. Functional studies demonstrated increased mitochondrial mass and activity in the resistant cell lines. In addition, we found that PTEN-induced kinase 1 (*PINK1*) was among the top upregulated genes in pathways associated with OxPhos, mitochondrial electron transport from NADH to ubiquinone, and ATP synthesis–coupled electron transport. A functional knock-down of PINK1 resulted in decreased cellular growth in 2B-OlapR and LN-OlapR cells, as well as increased sensitivity to olaparib treatment. Furthermore, targeting the expression of PINK1 resulted in a reduction in mitochondrial health and activity. These findings suggest that PINK1 plays a crucial role in modulating mitochondrial activity that confers olaparib resistance.

## Materials and Methods

### Cell lines, equipment, and culture reagents

Prostate cancer cell lines LNCaP and C4-2B were obtained from the ATCC. As previously described by our group, 2B-OlapR and LN-OlapR cells were derived through chronic exposure to increasing doses of olaparib for 1 year (21). The olaparib–resistant cell line is subsequently resistant to clinically relevant PARP inhibitors. Cell lines were maintained in liquid nitrogen for long-term storage, to be thawed with use over time. Once thawed, cells were passaged around every 5 days. All cell lines were routinely tested for Mycoplasma contamination using Mycoplasma PCR Detection Kit (Applied Biological Materials, Cat. #: G238). All assays conducted with these cell lines were conducted within 3 months of resuscitation after cryopreservation. C4-2B cells were maintained with RPMI 1640 media supplemented with 10% FBS, 100 IU penicillin, and 0.1 mg/mL streptomycin. Resistant cell lines were maintained in parallel in supplemented RPMI media with 5 μmol/L olaparib added. All cell lines were maintained at 37°C in a humidified incubator with 5% carbon dioxide. Olaparib was purchased from Selleckchem (Cat. #: S1060) for culture conditioning and growth assays.

### RNA-seq and GSEA analysis

Cell lines were plated for 72 hours with the previously described supplemented RPMI 1640 media. Single replicate RNA samples from both groups were isolated from cells plated and then tested for purity and concentration using a NanoDrop spectrophotometer from Thermo Fisher Scientific (Cat. #: ND-2000c). Singlet RNA samples were then submitted to the UC Davis Comprehensive Cancer Center’s Genomics Shared Resource for RNA-seq analysis, as previously described (21). Principal component analysis was conducted on the read counts gene-level data for all genes/transcripts passing filtering (filtered on expression >0.1) in the raw data (Gene Expression Omnibus accession number: GSE249514). GSEA on RNA-seq data was performed on the desktop software provided by the Broad Institute with gene sets provided by the MSigDB (http://software.broadinstitute.org/gsea/index.jsp). Gene sets were utilized to discern differential expression in transcriptomic data. The Hallmark gene set collection was preliminarily used for GSEA, followed by specific gene sets downloaded from the MSigDB to be used in the GSEA desktop software (http://www.gsea-msigdb.org/gsea/index.jsp). The GSEA data reported include the normalized enrichment score, nominal *P* value, and family-wise error rate *P* value (*P* value). Individual gene expression data from RNA-seq in each set were extracted, and the most upregulated genes were visualized in a heatmap.

### qRT-PCR, antisense oligonucleotides, and antibodies

RNA was extracted from sensitive and resistant cell cultures with TRIzol reagent from Thermo Fisher Scientific (Cat. #: 15596026). qRT-PCR) was subsequently performed with qScript cDNA SuperMix from Quantabio (Cat. #: 95048). SsoFast EvaGreen Supermix was then utilized from Bio-Rad (CAT. #: 1725203) with PINK1 primers from Integrated DNA Technologies (IDT) for the following sequence (F: 5′ GCC​TCA​TCG​AGG​AAA​AAC​AGG 3′, R: 5′ GTC​TCG​TGT​CCA​ACG​GGT​C 3′). The sample results were reported as a function of actin-normalized expression, standardized to normal fold change compared with the control.

Antisense oligonucleotides (ASO) were ordered from IDT with sequences targeting PINK1 (5′-rGrUrUr CrUrG rGrArC rCrArG rCrUrA rCrUrG–3′, 5′-rArArU rArArU rUrCrA rGrUrA rGrCrU rGrGrU–3′) for effective knock-down of PINK1 expression. As a negative control, siNC oligonucleotides were utilized from IDC (Lot. #: 921238). For all assays, siPINK1 ASOs were supplemented in media at a concentration of 40 nmol/L for a minimum of 18 hours before the start of each experiment. Before supplementation, ASOs were encapsulated in RNAiMAX from Invitrogen (Cat. #: 13778150).

For Western blot analysis, antibodies were ordered from Santa Cruz Biotechnology targeting PINK1 (Cat. #: 518052) and from Cell Signaling Technology targeting GAPDH (Cat. #: 2118-14C10) as a loading control. Antibodies were used according to the manufacturers’ recommended starting dilution.

### Seahorse Mito Stress Test and glycolytic rate asssay

Cells were plated at a density of 100,000 cells/mL in 5 mL of supplemented media on a 60-mm dish, followed with siNC or siPINK1 treatment at 40 nmol/L for 2 days. The cells were replated to Agilent Seahorse XF Cell Culture Microplate (Cat. #: 100777-004) at a density of 260,000 cells/mL in 100 μL supplemented RPMI and incubated at 37°C with 5% carbon dioxide. The following day, the cells were subjected to a mitochondrial stress test using Seahorse XF Cell Mito Stress Test Kit from Agilent (Cat. #: 103015-100) according to the manufacturer’s instruction on a Seahorse XFe24 analyzer. Note, an overall decrease in mitochondrial function in all cells treated with ASOs and RNAiMAX is observable. In an effort to ensure accurate relative readings, comparisons were only performed between cells with the same experimental conditions and from the same experimental run.

Mito Stress Test Kit offers the ability to derive the relative health and activity of mitochondrial electron transport chain (ETC) members through chemical manipulation through pharmacologic supplementation. Injection of oligomycin (ETC complex 5 inhibitor), carbonyl cyanide-4 (trifluoromethoxy) phenylhydrazone (FCCP, a mitochondrial membrane uncoupling agent), rotenone (complex 1 inhibitor), and antimycin A (complex 3 inhibitor) throughout the assay allows for calculation based on differential oxygen consumption rate (OCR) readings. Glycolytic Rate Assay Kit (Agilent, Cat. #: 103344-100) measures the OCR and extracellular acidification rate and determines the proton efflux rate at basal state and after rotenone/antimycin A and 2-deoxy-D-glucose (a glycolysis inhibitor) therapy. Initial data were recorded and reported using Seahorse Wave Desktop Software. Microplate well content was also subjected to protein quantification by Pierce Bradford Plus Protein Assay reagent from Thermo Fisher Scientific (Cat. #: 23238) for post hoc normalization.

### MitoTracker

C4-2B and 2B-OlapR cell lines were plated at a density of 55,000 cells/mL in 500 μL on 4-well chamber slides from Thermo Fisher Scientific (Cat. #: 154453). The following day, cells were incubated with 75 nmol/L concentration of MitoTracker Green from Thermo Fisher Scientific (Cat. #: M7514) RPMI media supplemented with 2% FBS for 45 minutes. The media was then aspirated, and the cells were covered with a mounting medium with 4',6-diamidino-2-phenylindole (DAPI) from Millipore Sigma (Cat. #: DUO82040) for nuclear counterstaining before covering with microscope cover glass from Fisher Scientific (Cat. #: 12-545-F). The microscope slides were then transferred to a dark place to dry for a minimum of 20 minutes.

Microscope imaging was performed using a Keyence BZ-X810 imaging system. Fluorescent emission was captured at 405 and 488 nm with identical exposure times for C4-2B and 2B-OlapR groups. Total fluorescence was measured in ImageJ for both fluorescent channels. total cell fluorescence (TCF) was then derived in FIJI. TFC readings for 488 nm (MitoTracker) were divided by 405 nm (DAPI) nuclear counterstain readings, and the ratio of MitoTracker TCF to DAPI TCF was derived. The results reported are as a percentage of control (either C42B or siNC).

### ETC complex I enzyme activity

Parental C42B and derived 2B-OlapR cells were utilized for ETC complex 1 (ETCC1) protein extraction and activity analysis. Four million cells of each group were plated and incubated for 2 days before the start of extraction. After collection, protein quantification with Pierce Bradford Protein Assay from Thermo Fisher Scientific (Cat. #: 23238) enables normalization for samples for use of Complex 1 Enzyme Activity Microplate Assay Kit from Abcam (Cat. #: ab109721). According to the kit guidelines, diluted protein extracts were incubated for 3 hours in ETCC1-specific antibody precoated microplate wells before washing and initiating the assay. Through the addition of ETCC1 substrate NADH, as well as a NAD+ specific dye, the activity of complex one can then be derived via colorimetric readings at 450 nm. Differential activity between groups was derived by utilizing measurements taken at 300 and 2000 seconds to determine a rate over time. Relative activity was then calculated and standardized to the control group to be reported in figures.

### Cell growth assays

Olaparib-resistant cells were plated at a density of 40,000 cells per mL in 0.5 mL of supplemented RPMI media on 24-well plates (Cat. # 142475) from Thermo Fisher Scientific. The following day, oligonucleotides were added to wells for a concentration of 40 nmol/L. The day after, olaparib or DMSO control was added to the cell media and incubated for 2 days. The cells were then trypsinized and counted using a Z1 particle counter (Beckman Coulter), and counts were reported as a percent of the siNC and DMSO combination group.

### Statistical analysis and figure creation

GraphPad Prism 10.0.0 was utilized for data normalization analysis. All data were subjected to preliminary statistical testing to ensure quality. Initially, data were analyzed for outliers using the ROUT test (*q* = 1%), followed by testing for variance and normality to ensure appropriate group testing conditions. Groups were compared using parametric *t* tests or ANOVAs when applicable. All experiments were repeated three times, and the representative mean results are reported, along with the SD, except for qPCR results in which the SEM is reported. For significance, *, *P* ≤ 0.05; **, *P* ≤ 0.01; ***, *P* ≤ 0.001; and ****, *P* ≤ 0.0001. The survival curve was created in R studio with “survival” and “survminer” R packages. Kaplan–Meier curves were plotted to analyze the survival probability of the two groups. A log-rank test was used to compare the overall survival or disease-free survival between the two groups of different gene expression levels. A *P* value lower than 0.05 indicated that the two groups differed significantly in overall survival or disease-free survival. All statistical analyses as well as most representative figures included were created using GraphPad PRISM desktop software. GSEA figures and statistical reporting were derived directly from the Broad Institute’s GSEA desktop software output. Heatmap generation consisted of log_2_ transformation of expression counts for naïve and resistant cell lines. Mean expression was computed between lines and normalized to that number for comparison of the two groups, with color corresponding to log_2_ fold change with “pheatmap” package in R studio.

### Data availability

Transcriptomic data can be accessed with the Gene Expression Omnibus accession number GSE249514. All other data collected in this research can be obtained by reaching out to the corresponding author A.C. Gao upon request.

## Results

### Olaparib-resistant prostate cancer cells exhibit enrichment of mitochondrial activity/OxPhos–related genes

To uncover key differences between olaparib-resistant and -naïve prostate cancer cell groups, we started with a broad characterization of the respective transcriptomes. Total RNA from resistant and sensitive cell lines were subjected to RNA-seq. Analysis was conducted utilizing gene sets that have been deposited in the Broad Institute’s collection from contributors Gene Ontology (geneontology.org) and the MSigDB (22).

**Figure 1:**
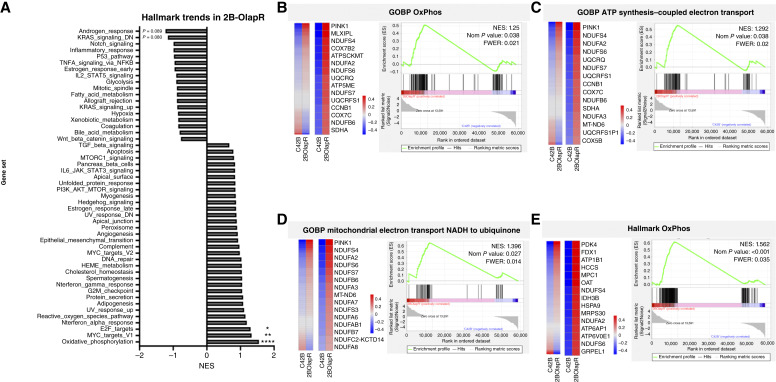
Gene enrichment in olaparib-resistant derived C4-2B cells (2B-OlapR) related to OxPhos. **A,** Hallmark collection gene sets in 2B-OlapR cells, with the OxPhos gene set the most enriched. **B–E,** Gene set expression heatmap for full set of the genes (left) in parental and resistant 2B cells, with the top 15 genes listed (right), and GSEA plots with summary statistics for the following: GOBP OxPhos (**B**), GOBP ATP synthesis coupled electron transport (**C**), GOBP Mitochondrial Electron Transport NADH to Ubiquinone (**D**), and the Hallmark OxPhos (**E**) from the MSigDB. FWER, family-wise error rate; NES, normalized enrichment score.

GSEA analysis utilizing the Hallmark gene set collection revealed enrichment in several general gene sets across the various cell lines. Among the most enriched Hallmark gene sets reported in the olaparib-resistant cell lines was the set encompassing genes associated with OxPhos (Fig. 1A; Supplementary Fig. S1A). Following initial collection screening, mitochondrial function– and OxPhos-based gene sets provided by Gene Ontology (https://geneontology.org/) were subsequently used to further validate the said enrichment (Fig. 1B-E; Supplementary Fig. S1B).

### Olaparib-resistant cells display increased mitochondrial functionality

**Figure 2:**
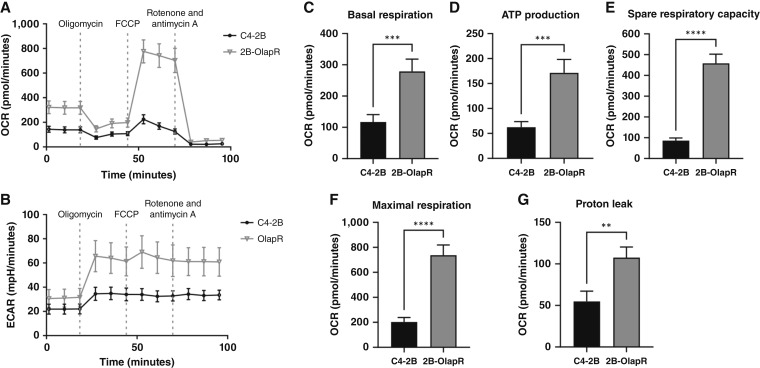
Seahorse Mito Stress Test assay results comparing C4-2B and 2B-OlapR cells at basal state and with mitochondrial inhibitor supplementation to deduce relative function. **A** and **B,** Mito Stress Test summary graphic representing differential OCR and extracellular acidification rate between naïve and resistant cell lines under normal conditions and with the addition of mitochondrial inhibitors. **C,** Cellular OCR at basal state is increased in the olaparib-resistant cell line. **D,** Amount of oxygen consumption utilized for ATP production, derived through OCR difference before and after oligomycin (ETC complex V inhibitor) is added. 2B-OlapR displays increased oxygen consumption related to ATP production. **E,** Change in the OCR between basal readings and maximal readings after FCCP (mitochondrial membrane uncoupler) is added. 2B-OlapR cells exhibit greater ability to increase respiration under mitochondrial insult. **F,** Difference in cellular OCR reading after rotenone and antimycin A (complex I and III inhibitors) are added, eliminating all ETC-based oxygen consumption. 2B-OlapR cells show amplified maximal respiration compared with parental C4-2B. **G,** Difference between readings after oligomycin addition and the readings after antimycin A/rotenone addition, inferring oxygen consumption not ultimately utilized for ATP production. The rate in 2B-OlapR cells is almost twice that in C4-2B. ECAR, extracellular acidification rate. **, P≤0.01; ***, P≤0.001; ****, P≤0.0001.

Having demonstrated enhanced activation of mitochondrial signaling pathways in olaparib-resistant cells, we next attempted to compare mitochondrial function between these two cell lines using Mito Stress Test Kit in a Seahorse XF analyzer. Mito Stress Test Kit offers a comprehensive analysis of major complexes in mitochondrial respiration through pharmacologic inhibition paired with observation of OCR and extracellular acidification rate. The resultant changes enable elucidation of the comparative activity of each participant (Fig. 2A and B; Supplementary Fig. S2A and S2B). Basal respiration was increased in resistant cells, with 2B-OlapR displaying a rate of more than two-fold the parental line C4-2B (Fig. 2C; Supplementary Fig. S2C), indicating a higher amount of mitochondrial activity at basal state prior to pharmacologic intervention. After initial readings, oligomycin is injected into microwells. Oligomycin acts as a complex 5 (ATP synthase) inhibitor, effectively halting the flow of protons out of the mitochondrial intermembrane space. This blockage halts ETC flow, and the change in the OCR infers oxygen consumption that is associated directly with the production of ATP from the ETC. As shown in (Fig. 2D), ATP production between 2B-OlapR and C4-2B lines differed, with 2B-OlapR displaying significantly higher ATP production. LNCaP background cell lines also showed trends of increase in the resistant background (Supplementary Fig. S2D). The second compound injected into the microwells is FCCP, which acts as a mitochondrial membrane uncoupling agent. The subsequent loss of proton gradient directs maximal ETC function and enables deduction of spare cellular respiration capacity. Spare capacity also enables functionality under great energy demands or cellular stress (such as FCCP supplementation). Olaparib-resistant cells had increased spare respiratory capacity compared with the parental line (Fig. 2E; Supplementary Fig. S2E). The final stage of the Mito Stress Test assay involved injection of both rotenone and antimycin A, inhibitors of ETC complexes 1 and 3, respectively, which abrogates ETC function and resultant oxygen consumption. The difference in values before and after rotenone/antimycin A addition reports maximal respiration, in other words, basal respiration + spare capacity. As shown in Fig. 2F, maximal respiration differed between 2B-OlapR and C4-2B, with 2B-OlapR displaying a much greater overall maximal respiration rate. A similar trend was seen in maximal respiration between LN-OlapR cells and LNCaP cells (Supplementary Fig. S2F).

Additional calculations that can be made to infer distinctions in metabolism reported by the Mito Stress Test assay is the subtraction of the OCR associated with ATP production from the OCR at basal respiration. The resultant leftover oxygen consumption encapsulates the OCR that is not being harnessed by the cell for ATP production, and so a rate of proton leak is inferred. Proton leak in treatment-resistant cells was around twice the amount observed in the treatment-naïve background (Fig. 2G; Supplementary Fig. S2G), suggesting a higher degree of mitochondrial plasticity and adaptability in 2B-OlapR cells, which can be crucial for cells under stress from olaparib treatment by restructuring mitochondrial dynamics and biogenesis to optimize survival. Collectively, using Mito Stress Test in a Seahorse analyzer, olaparib-resistant prostate cancer cells display enhanced mitochondrial functional activity.

### 2B-OlapR cells exhibit increased mitochondrial mass and higher mitochondrial complex 1 activity

**Figure 3:**
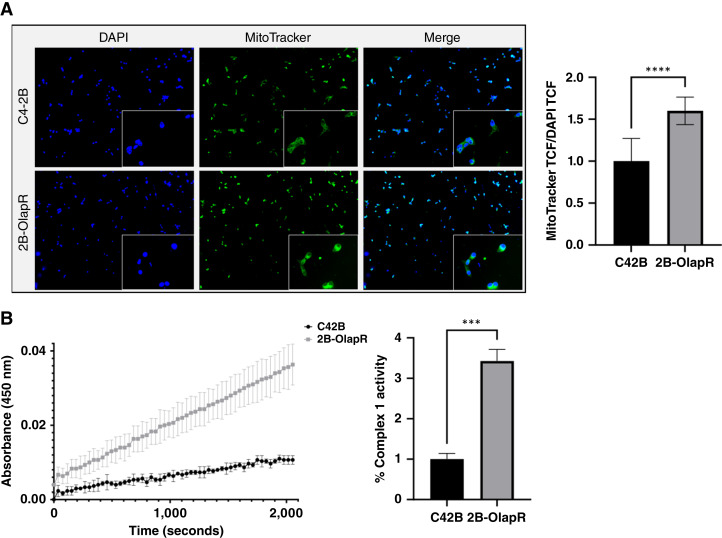
Mitochondrial live stain and ETC functional test demonstrate higher function in 2B-OlapR cells. **A,** Left, MitoTracker staining counterstained with nuclear-specific DAPI reveals higher amounts of relative mitochondrial mass in 2B-OlapR cells over C42B. 10× images shown, with 20× crops within. Right, TCF ratio of MitoTracker to DAPI displaying quantification of relative increased active mitochondria in the resistant cell subline. **B,** Left, ETCC1 kit assay results express a higher rate of ETCC1 activity in 2B-OlapR cells. Right, Reported rate of change in NAD+ associated absorbance between 300 and 2,000 seconds show significant increased ETCC1 rate in 2B-OlapR cells. ***, P≤0.001; ****, P≤0.0001.

To determine mitochondrial activity in living cells, we plated 2B-OlapR and C4-2B cells in a 4-well slide chamber overnight for adherence. The cells were thereafter stained with MitoTracker, which will passively diffuse and accumulate in active mitochondria. Utilizing a nuclear counterstain (DAPI), a ratio of nuclear to active mitochondrial TCF was derived and reported. The 2B-OlapR cell line displayed an increased quantity of active mitochondrial mass compared with the C4-2B cell line (Fig. 3A).

RNA-seq data analysis showed that the mitochondrial ETC complex gene signal pathway is significantly higher in 2B-OlapR cells compared with C4-2B cells (Fig. 1C and D; Supplementary Fig. S1C and S1D). Thus, we determined mitochondrial complex 1 activity in both 2B-OlapR and C4-2B cells. Among the complexes that occupy and contribute to the proton gradient to be utilized for ATP synthesis on the inner mitochondrial membrane is complex 1, also known as NADH ubiquinone oxidoreductase (23). The ETCC1 activity kit (Abcam) was used to determine relative complex 1 activity through incubation of cellular homogenates on a surface precoated with capture antibodies specific for complex 1. C4-2B and 2B-OlapR cells were collected and used, and levels of NADH dehydrogenation were recorded through the provided colorimetric dye that activates in the presence of NAD+ molecules. Differential changes in colorimetric readings over time between cell groups were uncovered. Indeed, 2B-OlapR complex 1 activity was observed to be more than 3 times greater than the activity of its parental C4-2B counterpart (Fig. 3B).

### PINK1 is overexpressed in 2B-OlapR cells

**Figure 4:**
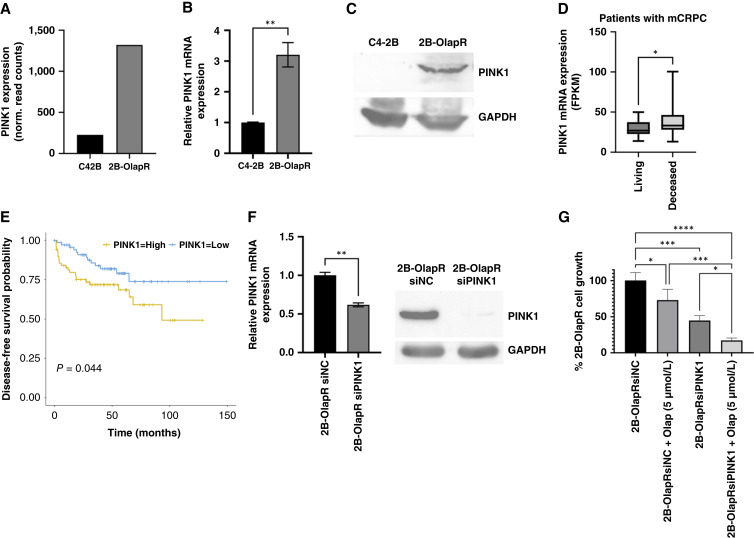
PINK1 expression is increased in 2B-OlapR cells and associated with negative patient prognosis. Treating 2B-OlapR cells with siPINK1 ASOs decreases PINK1 expression and protein level, as well as cell growth, and improves olaparib efficacy. **A,** Transcriptomic data reveal increased expression of PINK1 in the 2b-OlapR subline. **B,** qRT-PCR verification of increased *PINK1* gene expression in 2b-OlapR cells. **C,** Western immunoblot analysis showing increased cellular PINK1 protein in 2b-OlapR cells. **D,** Deceased patients with prostate cancer have higher amounts of tumor PINK1 expression (Abida 2019). **E,** High-PINK1–expressing striated patients with prostate cancer show lower survival over time (GSE21032). **F,** 2B-OlapR cells treated with siPINK1 show lower mRNA expression through qRT-PCR (left) and decrease in cellular protein (right). **G,** siPINK1 treatment decreases growth of 2B-OlapR cells and increases relative olaparib efficacy at 5 μmol/L. *, P≤0.05; **, P≤0.01; ***, P≤0.001; ****, P≤0.0001.

To determine the potential mechanisms which contribute to the enhanced mitochondrial activity in olaparib-resistant cells, we analyzed the transcriptomic data for parental and resistant cells in both 2B and LN backgrounds. *PINK1* was among the top upregulated genes in pathways associated with OxPhos, mitochondrial electron transport from NADH to ubiquinone, and ATP synthesis–coupled electron transport for resistant cells (Fig. 1B; Supplementary Fig. S1B). RNA-seq data showed that resistant cells exhibited an increase of PINK1 mRNA expression in resistant cells for both the 2B and LN sublines (Fig. 4A; Supplementary Fig. S3A). Following this initial screen, with higher PINK1 expression was confirmed by qRT-PCR in resistant cells (Fig. 4B; Supplementary Fig. S3B), as well as increased PINK1 protein was observed in 2B-OlapR cells (Fig. 4C).

Data analysis showed that PINK1 expression is significantly increased in deceased group versus living group in patients with metastatic prostate adenocarcinoma (Fig. 4D; ref. 23). Survival of patients when stratified by PINK1 expression shows significant differential outcomes, with higher PINK1 expression associated with poor survival probability (Fig. 4E; ref. 24; GSE21032). We next interrogated the effect of decreased expression of PINK1 on cellular viability. Through supplementation of siPINK1 over 2 days, both PINK1 mRNA expression and protein expression were reduced (Fig. 4F; Supplementary Fig. S3C). Resistant line cell growth was significantly decreased with siPINK1 treatment, and the growth was further decreased by adding olaparib (5 μmol/L; Fig. 4G; Supplementary Fig. S3D). Collectively, these data suggest that PINK1 is overexpressed in olaparib-resistant cells and is associated with poor survival in advanced prostate cancer and that inhibition of PINK1 decreased cell growth and resensitized olaparib treatment.

### Targeting PINK1 expression reduces mitochondrial functionality and mass and mitochondrial complex 1 activity

**Figure 5:**
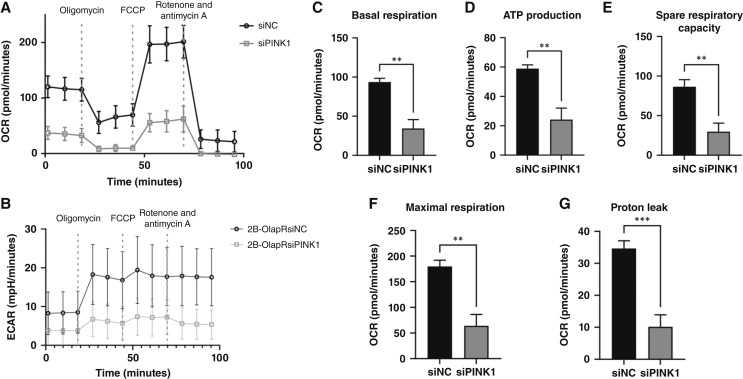
Seahorse Mito Stress Test assay results comparing 2B-OlapR cells with and without siPINK treatment at basal state and with mitochondrially relevant pharmacologic supplementation to deduce relative function convey decreased function after PINK1 expression ablation. **A** and **B,** Mito Stress Test summary graphic representing differential OCR and extracellular acidification rate between nonspecific and siPINK1-targeted OlapR cells. **C,** Cellular OCR at basal state is greatly reduced with PINK1 excision. **D,** ATP production–associated oxygen consumption is markedly reduced in 2B-OlapR cells treated with siPINK1. **E** and **F,** PINK1 expression reduction reveals consequential decreased spare capacity for respiration, as well as lessened maximal rate of ETC-based oxygen consumption. **G,** Decreasing PINK1 levels leads to less proton leakage out of the inter-mitochondrial membrane space inferred by OCR reading differences after oligomycin addition and after rotenone and antimycin addition. ECAR, extracellular acidification rate. **, P≤0.01; ***, P≤0.001.

**Figure 6:**
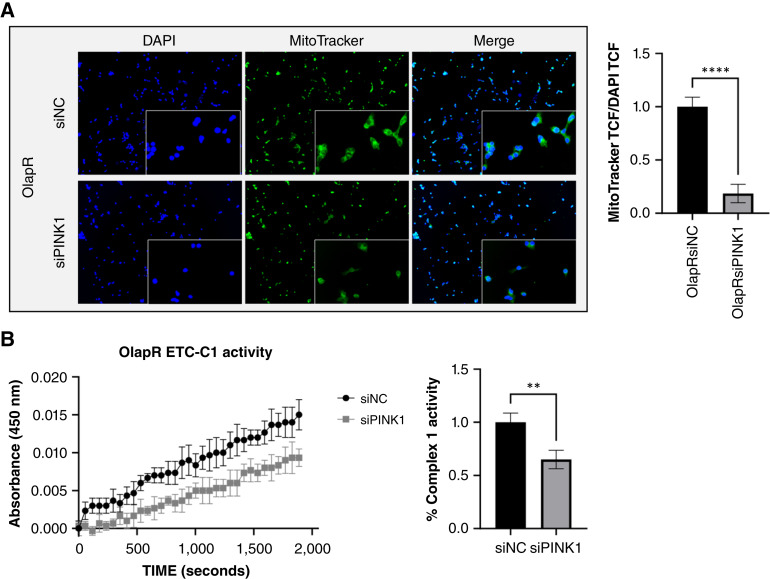
Inhibiting PINK1 expression decreases relative active mitochondria and lowers ETCC1 activity. **A,** Left, 2B-OlapR cells treated with siPINK1 show lower relative positive MitoTracker staining. 10× images shown, with 20× crops within. Right, MitoTracker to DAPI TCF ratio is reduced in the siPINK1 group. **B,** Left, Decreased PINK1 expression results in lower ETCC1 activity. Right, siPINK1-dosed 2B-OlapR cells report decreased the ETCC1 activity rate. **, P≤0.01; ****, P≤0.0001.

We next determined if targeting PINK1 could affect mitochondrial activity. We used Mito Stress Test kit with 2B-OlapR cells treated with siPINK1 to measure comparative mitochondrial function. Overall reduction in mitochondrial function was observed in 2B-OlapR cells treated with siPINK1 (Fig. 5A and B). Basal respiration was significantly reduced in the PINK1-targeted group (Fig. 5C). In addition to respiration at basal state, oxygen consumption associated with ATP production similarly decreased in the siPINK1-treated group (Fig. 5D). With FCCP addition, the rate of oxygen consumption increased significantly more in the nontreated group (Fig. 5E), and maximal respiration was observed to be considerably lower in the PINK1-targeted cell group (Fig. 5F), as well as proton leak (Fig. 5G).We demonstrated that knock-down of PINK1 expression decreased mitochondria mass in 2B-OlapR cells, as shown by MitoTracker fluorescent staining (Fig. 6A). Furthermore, diminished PINK1 expression reduced mitochondrial complex I activity (Fig. 6B). Collectively, these results indicated that mitochondrial functionality was reduced by blocking PINK1 expression in 2B-OlapR cells.

## Discussion

In this study, we evaluated the mitochondrial activity linked to olaparib resistance in castration-resistant and -sensitive prostate cancer cells. We found that OxPhos emerged as one of the most enriched gene sets in resistant sublines, associated with the production and maintenance of mitochondria. Several functional assays confirmed an increase in mitochondrial mass and activity in the resistant cells. Notably, *PINK1* was among the most upregulated genes in pathways linked to OxPhos, mitochondrial electron transport from NADH to ubiquinone, and ATP synthesis via electron transport. Knocking down PINK1 not only reduced cellular growth but also heightened sensitivity to olaparib. Additionally, targeting PINK1 expression decreased mitochondrial function and activity. These results underscore the critical role of PINK1 in regulating mitochondrial activity, thereby contributing to olaparib resistance.

OxPhos is a metabolic pathway conducted in functional mitochondria, which utilizes a series of protein complexes (ETC complexes) in its interior membrane. These ETC complexes utilize products of the tricarboxylic acid cycle, NADH, FADH2, and succinate as electron donors to effectively pump protons across this interior membrane into the inter-mitochondrial space, creating a gradient of protons (membrane potential) through the shuttling of electrons along the chain that is utilized by ETC complex 5 (ATP synthase) to produce ADP phosphorylation (23). Mitochondrial function and its products are not only important for cell survival but are also shown to be upregulated in various cancers (24). Increases in OxPhos have also been observed to drive viability in different cancer types, including prostate cancer (25, 26). Also, targeting mitochondrial function in drug-resistant cancers has been shown to resensitize cancer to therapy in various settings (27, 28). The GSEA, specifically using the Hallmark gene set collection, highlighted several gene sets that were differently enriched between olaparib-resistant and -naïve prostate cancer cell lines. Notably, the OxPhos gene set showed significant enrichment in the resistant cells compared with the parental cells, as illustrated in Fig. 1A and B. This initial finding suggested a potential upregulation of mitochondrial activity as a mechanism of resistance to olaparib supplementation. To further substantiate this observation, we used additional mitochondrial function– and OxPhos-related gene sets from Gene Ontology. These gene sets confirmed the initial enrichment observed, indicating a consistent upregulation of genes involved in mitochondrial function and OxPhos in the resistant cells. These findings suggest that the upregulation of mitochondrial and OxPhos pathways might be a crucial adaptive mechanism by which prostate cancer cells develop resistance to olaparib. This enhanced mitochondrial function could be pivotal for sustaining the energy demands of the resistant cells, highlighting potential targets for overcoming olaparib resistance in prostate cancer treatment.

Among the most upregulated genes identified across the gene sets utilized, the *PINK1* gene was identified as crucial for mitochondrial maintenance and activity (25). PINK1 is a 63KDa serine/threonine kinase that works as a regulator for multiple cellular functions, including mitochondrial health (26, 27). Specifically in cancer cells, PINK1 has previously been implicated not only in mitochondrial homeostasis but also in areas including cell-cycle progression, apoptosis, and chemotherapeutic efficacy (25, 28–30). PINK1 can monitor and signal for degradation of faulty mitochondria through complex interactions with outer and inner mitochondrial translocases which potentiates autophagic machinery recruitment in dysfunctional settings (31–34). Additionally, PINK1 has been reported to contribute to mitochondrial activity maintenance via activation of the AKT axis through phosphorylation of the Rictor subunit of mTORC2 (35, 36). PINK1 was observed to increase AKT activity in ovarian cancer cells as well and enhanced resistance to chemotherapy (37). In non–small cell lung cancer, reduction in PINK1 resulted in diminished cell proliferation and reduced cellular ATP production (38). In hepatocellular carcinoma, PINK1 has been reported to contribute to chemoresistance of multitarget tyrosine kinase inhibitors (30). Expression of PINK1 is reported to be essential in maintaining high mitochondrial activity described in drug-tolerant persistent lung adenocarcinoma cells, with PINK1 ablation resulting in decrease in OxPhos (39). Thus far, the effect of targeting PINK1 expression in the prostate cancer landscape is absent. Considering the implications in other cancer types regarding PINK1 and its positive association with mitochondrial activity and chemoresistance across multiple drug classes, the need for an examination in our specific model is evident.

In our resistant cells, we found increased amounts of PINK1 mRNA as well as protein compared with the parental counterparts. Downregulation of PINK1 mRNA was observed to decrease cellular PINK1 and caused a multitude of phenotypic changes in our model. Resistant cell growth and relative resistance were considerably decreased when PINK1 expression was targeted. Relative mitochondrial function, mass, and ETCC1 activity were also reduced upon PINK1 targeting. Additionally, worse clinical prognosis and lower survival status from the public databases were determined to be associated with abnormally upregulated PINK1 expression in two different prostate cancer cohorts. It is important to note that ATP is heavily relied upon for and promotes DNA repair (40, 41), which offers a resistance rationale for DNA damage heavily resistant cells (19, 21). It is also important to outline that olaparib itself has been reported to be an inhibitor of ETC 1, and its utility has been noted in targeting temozolomide-resistant glioblastoma through inhibiting OxPhos (42). Considering this literature, increased OxPhos expression and functionality in OlapR cells could be a direct mechanism of resistance to olaparib if olaparib is inhibiting OxPhos directly. Indeed, upon interrogation, very little change was observed for glycolytic pathway activation and glycolysis (Supplementary Figs. S4A–S4G and S5A–S5G). Instead, the olaparib-resistant cells exhibited a shift away from glycolysis and toward alternative means of proton efflux (Supplementary Figs. S4A–S4G and S5A–S5G). In summary, this study investigates the role of mitochondrial changes and the *PINK1* gene in olaparib resistance in CRPC cells, revealing that resistant cells exhibit enhanced mitochondrial functionality and increased expression of mitochondrial function– and OxPhos-related genes compared with naïve cells. Overexpression of PINK1 in 2B-OlapR and LN-OlapR cells correlates with increased resistance to olaparib and worse clinical outcomes in patients with prostate cancer . These findings suggest that targeting PINK1 could mitigate both mitochondrial function and olaparib resistance, offering a potential therapeutic target to combat olaparib resistance in prostate cancer.

## Supplementary Material

Figure S1 supplementary figure

Figure S2 supplementary data

Figure S3 supplementary data

Figure S4 supplementary data

Figure S5 Supplementary data
