# Supplementary material for: PINK1-Mediated Mitochondrial Activity Confers Olaparib Resistance in Prostate Cancer Cells
Source: Cancer Res Commun. 2024 Nov 20;4(11):2976–85. doi: 10.1158/2767-9764.CRC-24-0339 (PMC11577557; doi:10.1158/2767-9764.CRC-24-0339)
Supplement: Figure S1 — supplementary figure [file crc-24-0339_figure_s1_suppsf1.pdf]

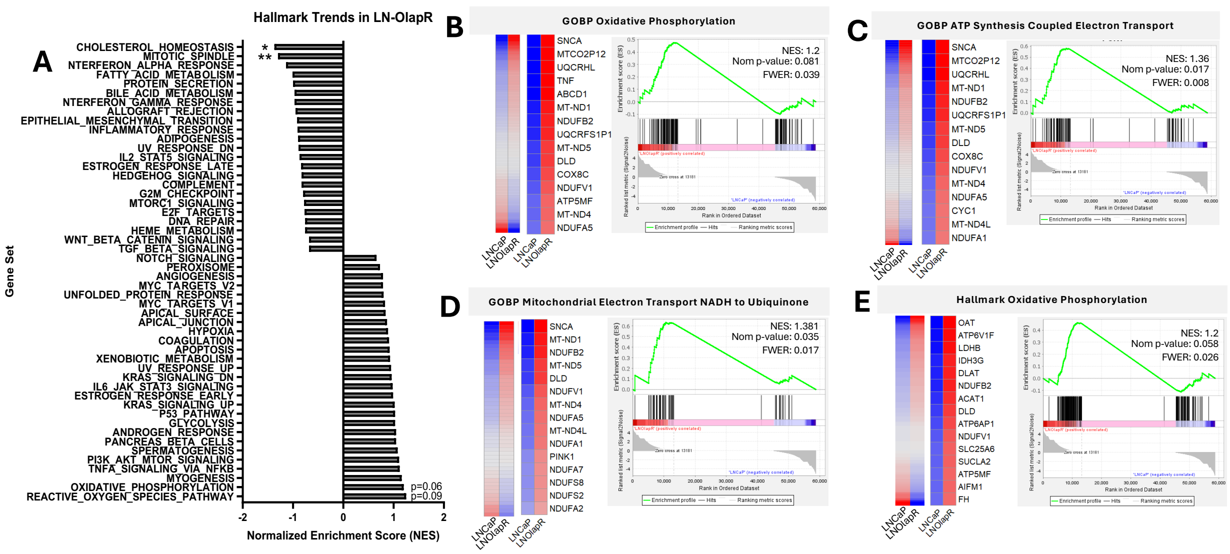

Figure S1: Gene Enrichment in Olaparib Resistant derived LNCap cells (LN-OlapR) related to oxidative phosphorylation.
A: Trends in Hallmark Collection Gene Sets in LN-OlapR cells, with the Oxidative Phosphorylation gene set the second most enriched. B-G: Gene set expression heatmap of full set of genes in parental and resistant LN cells, with top 15 genes listed and GSEA plots with summart stats for GOBP Oxidative Phosphorylation (B), GOBP ATP synthesis coupled electron transport (C), GOBP Mitochondrial Electron Transport NADH to Ubiquinone (D), and the Hallmark Oxidative Phosphorylation (E) gene sets from MSigDB.
