# Supplementary material for: PINK1-Mediated Mitochondrial Activity Confers Olaparib Resistance in Prostate Cancer Cells
Source: Cancer Res Commun. 2024 Nov 20;4(11):2976–85. doi: 10.1158/2767-9764.CRC-24-0339 (PMC11577557; doi:10.1158/2767-9764.CRC-24-0339)
Supplement: Figure S2 — supplementary data [file crc-24-0339_figure_s2_suppsf2.pdf]

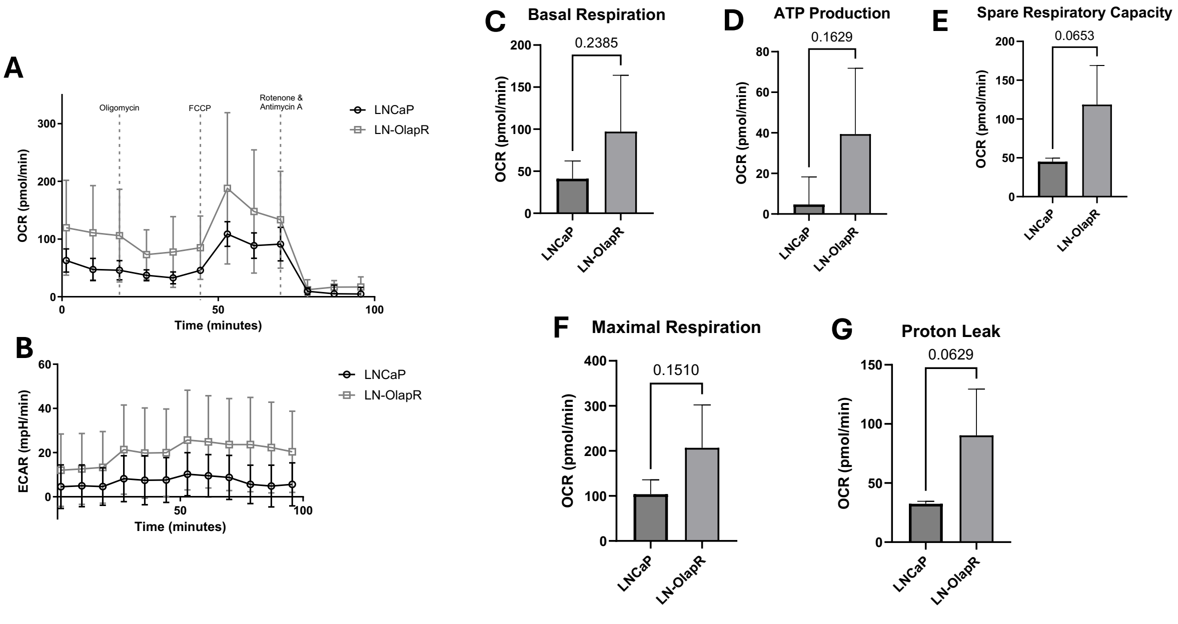

Figure S2: Seahorse Mito Stress Test assay results comparing LNCaP and LN-OlapR cells at basal state and with mitochondrially-relevant pharmacologic supplementation to deduce relative mitochondrial function.

A,B: Mito Stress Test summary graphic representing differential OCR and ECAR between naïve and resistant cell lines, with LN-OlapR displaying significantly increased basal oxygen consumption. C: Cellular oxygen consumption rate (OCR) at basal state is increased in Olaparib-resistant cell line. D: Amount of oxygen consumption utilized for ATP production, derived through OCR difference before and after Oligomycin (ETC Complex V inhibitor) is added. LN-OlapR displays increased oxygen consumption related to ATP production. E: Change in OCR between basal readings and max readings after FCCP (mitochondrial membrane uncoupler) is added. LN-OlapR cells exhibit greater ability to increase respiration under mitochondrial insult. F: Difference in cellular OCR reading after Rotenone and Antimycin A (Complex I and III inhibitors) are added, eliminating all ETC-based oxygen consumption. LN-OlapR cells show amplified maximal respiration compared to parental LNCaP. G: Difference between readings after oligomycin addition and the readings after antimycin A and Rotenone addition, inferring oxygen consumption not ultimately utilized for ATP production. The rate in LN-OlapR cells is around twice that of parental LNCaP.
