# Supplementary material for: PINK1-Mediated Mitochondrial Activity Confers Olaparib Resistance in Prostate Cancer Cells
Source: Cancer Res Commun. 2024 Nov 20;4(11):2976–85. doi: 10.1158/2767-9764.CRC-24-0339 (PMC11577557; doi:10.1158/2767-9764.CRC-24-0339)
Supplement: Figure S3 — supplementary data [file crc-24-0339_figure_s3_suppsf3.pdf]

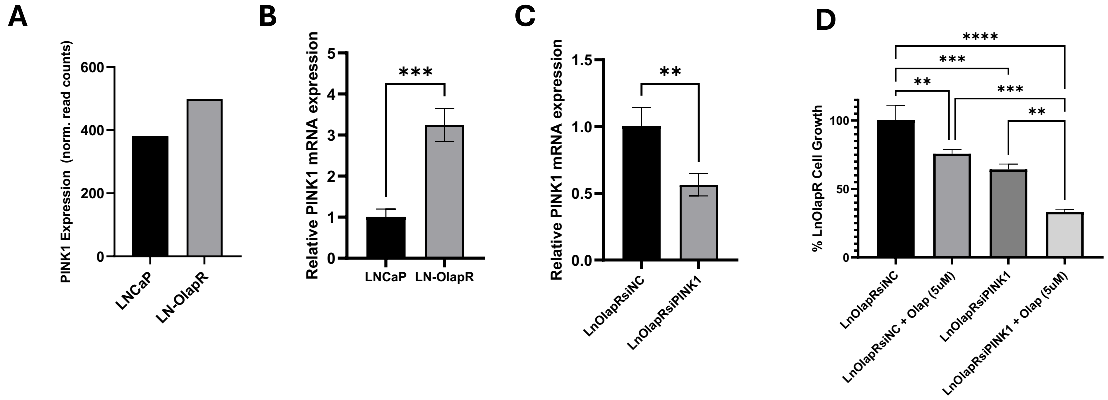


Figure S3: Treating LN-OlapR cells with siPINK1 antisense oligonucleotides (ASOs) decrease PINK1 expression, decreasing cell growth and improving Olaparib efficacy. A: Transcriptomic data reveals increased expression of PINK1 in LN-OlapR subline. B: RT-qPCR verifies increased PINK1 gene expression in LN-OlapR cells. C: LN-OlapR cells treated with siPINK1 show lower mRNA expression. D: siPINK1 treatment decreases growth of LN-OlapR cells, and increases relative olaparib efficacy at 5uM.
