# Supplementary material for: PINK1-Mediated Mitochondrial Activity Confers Olaparib Resistance in Prostate Cancer Cells
Source: Cancer Res Commun. 2024 Nov 20;4(11):2976–85. doi: 10.1158/2767-9764.CRC-24-0339 (PMC11577557; doi:10.1158/2767-9764.CRC-24-0339)
Supplement: Figure S4 — supplementary data [file crc-24-0339_figure_s4_suppsf4.pdf]

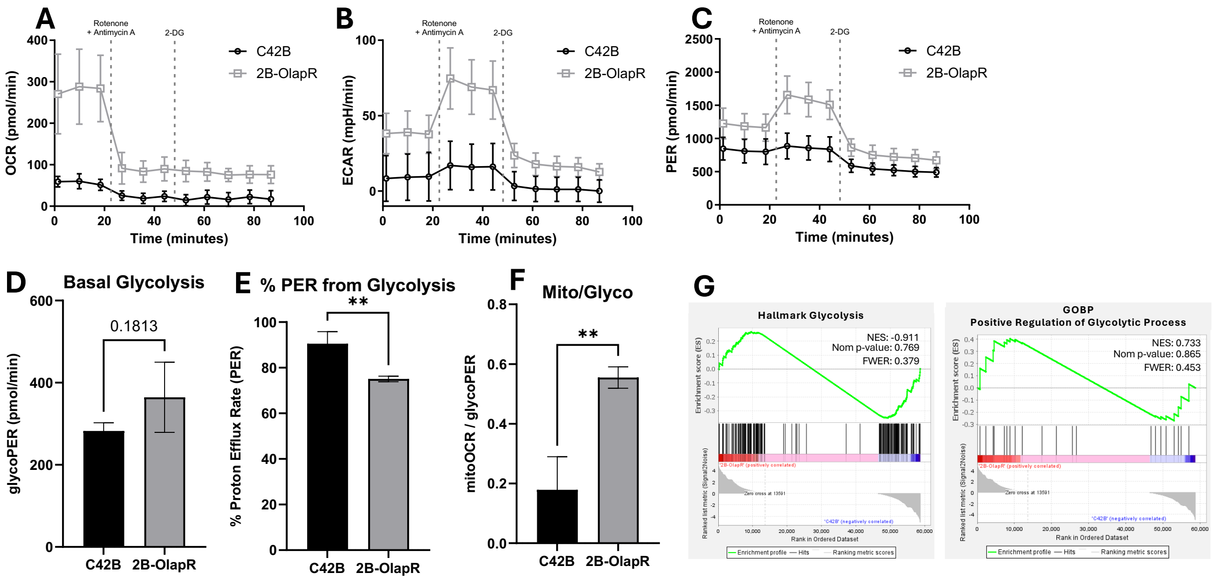

Figure S4: Seahorse Glycolytic Rate Assay Results and glycolysis related-GSEA results A,B,C: Oxygen Consumption Rate (OCR), Extracellular Acidification Rate (ECAR), and Proton Efflux Rate (PER) after additions of Rotenone/Antimycin-A and then 2-Deoxy-D-glucose (2-DG) in C42B and 2B-OlapR cells. D: Measurement of proton efflux dependent on glycolysis in naïve and resistant cells at basal state. E: Derived proportion of proton efflux resultant of glycolysis in C42B and 2B-OlapR cells. F: Ratio of mitochondrial OCR to glycolytic based PER in parental and resistant cells. G: GSEA of glycolysis and glycolytic process regulation in sensitive and resistant 2B cell subline from MSigDB.
